# Differential Modulation by IL-17A of Cholangitis versus Colitis in IL-2Rα Deleted Mice

**DOI:** 10.1371/journal.pone.0105351

**Published:** 2014-08-18

**Authors:** Wei Yang, Yuan Yao, Yan-Qing Yang, Fang-Ting Lu, Liang Li, Yin-Hu Wang, Takahiko Nakajima, Koichi Tsuneyama, William M. Ridgway, M. Eric Gershwin, Zhe-Xiong Lian

**Affiliations:** 1 Liver Immunology Laboratory, Institute of Immunology and CAS Key Laboratory of Innate Immunity and Chronic Disease, School of Life Sciences and Medical Center, University of Science and Technology of China, Hefei, China; 2 Department of Diagnostic Pathology, Graduate School of Medicine and Pharmaceutical Science for Research, University of Toyama, Toyama, Japan; 3 Division of Immunology, Allergy and Rheumatology, University of Cincinnati, Cincinnati, OH, United States of America; 4 Division of Rheumatology, Allergy and Clinical Immunology, University of California at Davis School of Medicine, Davis, CA, United States of America; 5 Innovation Center for Cell Biology, Hefei National Laboratory for Physical Sciences at Microscale, Hefei, China; University of Chicago, United States of America

## Abstract

IFN-γ is a signature Th1 cell associated cytokine critical for the inflammatory response in autoimmunity with both pro-inflammatory and potentially protective functions. IL-17A is the hallmark of T helper 17 (Th17) cell subsets, produced by γδT, CD8+ T, NK and NKT cells. We have taken advantage of our colony of IL-2Rα^−/−^ mice that spontaneously develop both autoimmune cholangitis and inflammatory bowel disease. In this model CD8+ T cells mediate biliary ductular damage, whereas CD4+ T cells mediate induction of colon-specific autoimmunity. Importantly, IL-2Rα^−/−^ mice have high levels of interferon γ (IFN-γ), and interleukin-17A (IL-17A). We produced unique double deletions of mice that were either IL-17A^−/−^IL-2Rα^−/−^ or IFN-γ^−/−^IL-2Rα^−/−^ to specifically address the precise role of these two cytokines in the natural history of autoimmune cholangitis and colitis. Of note, deletion of IL-17A in IL-2Rα^−/−^ mice led to more severe liver inflammation, but ameliorated colitis. In contrast, there were no significant changes in the immunopathology of double knock-out IFN-γ^−/−^ IL-2Rα^−/−^ mice, compared to single knock-out IL-2Rα^−/−^ mice with respect to cholangitis or colitis. Furthermore, there was a significant increase in pathogenetic CD8+ T cells in the liver of IL-17A^−/−^IL-2Rα^−/−^ mice. Our data suggest that while IL-17A plays a protective role in autoimmune cholangitis, it has a pro-inflammatory role in inflammatory bowel disease. These data take on particular significance in the potential use of anti-IL-17A therapy in humans with primary biliary cirrhosis.

## Introduction

Primary biliary cirrhosis (PBC) is a chronic autoimmune liver disease characterized by destruction of small bile ducts and the presence of anti-mitochondrial antibodies (AMA) [1]. We previously reported that IL-2Rα^−/−^ mice spontaneously develop an autoimmune biliary ductular disease, exhibiting major serological and histological characteristics of human PBC [2] as well as an inflammatory bowel disease (IBD), characterized by diarrhea and wasting [3]. By crossing IL-2Rα^−/−^ mice with CD4 knockout and CD8 knockout mice, we demonstrated that CD8+ T cells mediate biliary ductular damage whereas CD4+ T cells mediate induction of colon-specific autoimmunity [4]. Serum levels of inflammatory cytokines, including TNF-α, IL-12p40, and IL-6, increased with the age of animals. There are particularly high circulating levels of Th1 inflammatory cytokines, particularly IFN-γ and Th17 inflammatory, including IL-17A [2], [4]. IFN-γ is the signature Th1 cell associated cytokine, which plays a critical role in inflammation and autoimmune disease, with both proinflammatory and protective functions [5]. IL-17A is the hallmark cytokine of T helper 17 (Th17) cell subset, produced by γδT cells [6], CD8+ T cells [7], [8], natural killer (NK) cells [9], [10] and NKT cells [11], [12]. A pathogenetic role for the Th17 pathway has been established in models of colitis [13]. Furthermore, recent studies show that the frequency of IL-17+ cells is significantly elevated in a variety of chronic liver diseases including alcoholic liver disease, viral hepatitis and hepatocellular carcinoma [14].

To examine in specific detail the role of IFN-γ versus IL-17A in autoimmune cholangitis and colitis, we took advantage of IL-17A^−/−^IL-2Rα^−/−^ and IFN-γ^−/−^IL-2Rα^−/−^ mice. We report herein that deletion of IL-17A in IL-2Rα^−/−^ mice aggravated cholangitis but ameliorated colitis. In contrast, there was no significant effect of the deletion of IFN-γ on the immunopathology of either autoimmune cholangitis or colitis. Importantly, T cells, particularly CD8+ T cells, were significantly increased in IL-17A^−/−^IL-2Rα^−/−^ mice. Our data suggests that IL-17A plays a protective role in autoimmune cholangitis but a proinflammatory role in colitis in IL-2Rα^−/−^ mice.

## Materials and Methods

### Mice

IL-2Rα^−/−^ (B6;129S4-Il2ra^tm1Dw/J^) and IFN-γ^−/−^ (B6.129S7-IFN-γ^tm1Ts^) mice on a C57B/6J background were initially obtained from the Jackson Laboratory (Bar Harbor, Maine, USA). IL-17A^−/−^ mice were donated by Dr. Yoichiro Iwakura (University of Tokyo, Tokyo, Japan). All genetically modified mice studied herein have been backcrossed to C57BL/6 background for at least 10 generations. IL-2Rα^−/−^ were bred to heterozygosity to these strains. To generate IL-17A^−/−^IL-2Rα^−/−^ and IFN-γ^−/−^IL-2Rα^−/−^ mice, IL-17A^−/−^ or IFN-γ^−/−^ mice were mated with IL-2Rα^+/−^ mice to obtain IL-17A^+/−^IL-2Rα^+/−^ or IFN-γ^+/−^IL-2Rα^+/−^ mice, which were subsequently backcrossed with IL-17A^−/−^ or IFN-γ^−/−^ mice to obtain IL-17A^−/−^IL-2Rα^+/−^ or IFN-γ^−/−^IL-2Rα^+/−^ mice; IL-17A^−/−^IL-2Rα^−/−^ or IFN-γ^−/−^IL-2Rα^−/−^ mice were obtained by inter-breeding. The IL-2Rα gene was identified by flow cytometric analysis based on mean fluorescent intensity of CD25. All mice were studied between 12 and 16 weeks of age and animals were individually housed in ventilated cages under the specific pathogen-free conditions. All animal experimental protocols were approved by the Institutional Animal Care and Use Committee of the School of Life Sciences, University of Science and Technology of China, Hefei, China. Animals were anesthetized for blood collection with Xylazine (5 mg/kg)/Ketamine (25 mg/kg) administered subcutaneously. Euthanasia was performed by an overdose of CO_2_ by inhalation, consistent with the recommendations on the Panel on Euthanasia of the American Veterinary Medical Association. There was no surgery performed and any animals exhibiting discomfort or distress by the veterinary staff were euthanized.

### Flow cytometry

Lymphocytes from spleen and liver were isolated using 40%/70% percoll (GE Healthcare, Little Chalfont, United Kingdom) [15]. Mesenteric lymph nodes were disrupted between two glass slides, suspended in PBS and passed through a nylon mesh; mononuclear cells were collected by centrifugation 800 g for 5 min. To detect subsets, cell preparations were incubated with anti-mouse FcR blocking reagent (Biolegend, San Diego, CA) and then incubated at 4°C for 10 minutes. Lymphocytes were then stained for 20 minutes with an optimal combination of fluorochrome-conjugated antibodies, including anti-CD44 FITC, anti-CD8β PE, anti-CD62L PerCP/CY5.5, anti-CD3 Alexa Fluora 647, anti-CD19 APC/CY7, anti-NK1.1 PE/CY7, anti-CD4 Pacific Blue, anti-CD8a V500. All antibodies were purchased from Biolegend (San Diego, CA) except for anti-CD8a V500 (BD Biosciences, San Jose, California). Stained cells were washed with PBS/BSA. For intracellular cytokine staining, cells were re-suspended in 10% FBS RPMI and stimulated with Cell Stimulation Cocktail (plus protein transport inhibitors) (eBioscience, San Diego, CA, USA) at 37°C for 4 hours with 5% CO_2_. Cells were then stained for surface markers using anti-CD4 PerCP/CY5.5, anti-NK1.1 PE/CY7, anti-CD3 Pacific Blue and anti-CD8β FITC (Biolegend), then intracellular stained with anti-IFN-γ PE and anti-IL-17A APC (Biolegend) after fixation and permeabilization with Fixation Buffer and Permeabilization Wash Buffer (Biolegend), Rat IgG1, κ PE and Rat IgG1, κ APC were used as isotype controls for anti-IFN-γ PE and anti-IL-17A APC respectively. Finally, samples were subjected to multiple-color analysis by BD FACSVerse flow cytometer (BD Biosciences). Acquired data were analyzed with FlowJo Software (Tree Star, Inc, Ashland, USA).

### Histopathology

Liver and colon were fixed in 4% paraformaldehyde, embedded in paraffin and cut into 4 and 6 µm sections respectively, then deparaffinized, stained with hematoxylin and eosin (H&E), and evaluated using light microscopy. The quantitation of tissue damage was performed by a “blinded” pathologist. Firstly, the degree of portal inflammation was evaluated and scored according to the most severe lesions as follows: 0, no change; 1, minimal inflammation; 2, mild inflammation; 3, moderate inflammation; 4, severe inflammation. In addition, the degree of inflammatory frequency in a specimen was determined by the percentage of affected tissue within the total hepatic lobules per specimen and coded as follows: 0, none, 1, 1%–10%; 2, 11–20%; 3, 21–50%; 4, more than 50%. Finally, a summary score that includes severity and frequency analysis was generated as the sum of these scores. We note that lobular inflammation was scored in the same fashion as portal inflammation. Second, bile duct damage was evaluated firstly by the degree of severity in the most severe lesions as follows: 0, no change; 1, epithelial damage (only cytoplasmic change); 2, epithelial damage with cytoplasmic and nuclear change; 3, non-suppurative destructive cholangitis (CNSDC); 4, bile duct loss. The frequency of bile duct damage was then scored as follows: 0, none; 1, 1%–10%; 2, 11–20%; 3, 21–50%; 4, more than 50%. To evaluate bile duct loss, bile duct epithelium was highlighted by immunostaining using rabbit anti-keratin wide antibody (DAKO, Glostrup, Denmark). To obtain an integrative evaluation, the scores of severity and frequency were added together. Third, colon histopathology was scored as follows: 0, no significant changes; 1, minimal scattered mucosal inflammatory cell infiltrates, with or without minimal epithelial hyperplasia; 2, mild scattered to diffuse inflammatory cell infiltrates, sometimes extending into the submucosa and associated with erosions, with mild to moderate epithelial hyperplasia and mild to moderate mucin depletion from goblet cells; 3, moderate inflammatory cell infiltrates that were sometimes transmural, with moderate to severe epithelial hyperplasia and mucin depletion; and 4, marked inflammatory cell infiltrates that were often transmural and associated with crypt abscesses and occasional ulceration, with marked epithelial hyperplasia, mucin depletion, and loss of intestinal glands [16].

### Quantitative PCR

Total RNA from liver and colon was extracted with RNeasy Mini Kit (Qiagen, Germany), and cDNA synthesized with the PrimeScript RT reagent Kit (Takara, Dalian, China). Quantitative PCR was performed using a SYBR Premix Ex TaqTM II (Takara, Dalian, China). Data were collected by an ABI StepOne real-time PCR system (Applied Biosystems, Carlsbad, CA). The PCR primers used in this study are listed in Table 1. The expression levels of target genes were normalized to the housekeeping gene Gapdh (ΔCt), and the results calculated by 2^−ΔΔCt^ method [17].

**Table 1:** Real Time PCR primers used in this study.

Genes	Forward (5′-3′)	Reverse (5′-3′)
*IFNγ*	TAGCCAAGACTGTGATTGCGG	AGACATCTCCTCCCATCAGCAG
*IL6*	CCACTTCACAAGTCGGAGGCTTA	CCAGTTTGGTAGCATCCATCATTTC
*IL7*	CTTGTTCTGCTGCCTGTCAC	CTTGCGAGCAGCACGATTTAG
*IL10*	GCCAGAGCCACATGCTCCTA	GATAAGGCTTGGCAACCCAAGTAA
*IL15*	CATCCATCTCGTGCTACTTGTGTT	CATCTATCCAGTTGGCCTCTGTTT
*IL-17a*	ACTACCTCAACCGTTCCACG	TTCCCTCCGCATTGACACAG
*IL18*	CAGGCCTGACATCTTCTGCAA	TCTGACATGGCAGCCATTGT
*IL21*	CTTCGTCACCTTATTGACATTGTTG	CCAGGGTTTGATGGCTTGA
*IL22*	GGTGACGACCAGAACATCCA	GACGTTAGCTTCTCACTTTCCTT
*IL27p28*	TCGATTGCCAGGAGTGAACC	AAGTGTGGTAGCGAGGAAGC
*Ebi3*	GGAACAGAGCCACAGAGCAT	AGAGCCACGAGAGCTGTTTC
*TNFα*	AAGCCTGTAGCCCACGTCGTA	AGGTACAACCCATCGGCTGG
*Gapdh*	CATGGCCTTCCGTGTTCCTA	CCTGCTTCACCACCTTCTTGAT

### Serum Cytokines

The levels of IFN-γ, tumor necrosis factor α (TNF-α), IL-2, IL-4, IL-6 and IL-10 from serum were measured simultaneously with a cytometric bead array kit (Mouse Th1/Th2/Th17 CBA kit, BD Biosciences), using a FACSVerse flow cytometer with CBA software (BD Biosciences).

### Statistical Analysis

Data are presented as the mean ± standard error. The histological scores of liver inflammation and colitis were compared using one-way analysis of variance (ANOVA) followed by Kruskal-Wallis multiple comparisons. The two-tailed unpaired Mann-Whitney test was used in other comparisons. A value of p<0.05 was considered statistically significant; a value of 0.05<p<0.1 was noted as only a trend.

## Results

### IL-2Rα^−/−^ CD4+ T cells are the primary source of IL-17A; IL-2Rα^−/−^ CD8+ T cells predominantly produce IFN-γ

**Figure 1:**
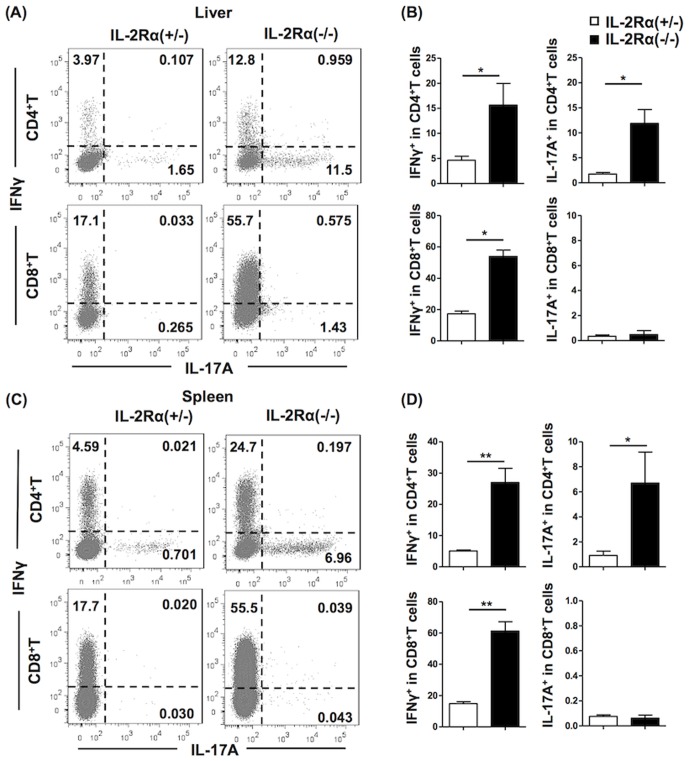
Cytokine profiles of CD4+ and CD8+ T cells in the liver and spleen of IL-2Rα^−/−^ mice and IL-2Rα^+/−^ mice. Hepatic and splenic MNC were analyzed by flow cytometry. Flow cytometry profiles are shown in (A) and (C), and quantification of frequency of IFN-γ+ and IL-17A+ cells in the CD4+ and CD8+ T cell populations in (B) and (D). Data are representative with four mice in IL-2Rα^−/−^ group and five mice in IL-2Rα^+/−^ group. * P<0.05, ** P<0.01.

We determined the cellular source of IL-17A and IFN-γ in IL-2Rα^−/−^ mice. CD8+ T cells from IL-2Rα^−/−^ mice produced a greater quantity of IFN-γ compared to IL-2Rα^+/−^ in liver (*P* = 0.0159), but almost no IL-17A. In contrast, IL-2Rα^−/−^ CD4+T cells produced more IL-17A than IL-2Rα^+/−^ CD4+ T cells (*P* = 0.0159), while CD4+T cells also produced some IFN-γ but at levels lower than CD8+T cells (*P* = 0.0286) (Figure 1A and B). Similar results were found in splenic CD4+T cells expressing intracellular IL-17A (*P* = 0.0190) and CD8+T cells expressing intracellular IFN-γ (*P* = 0.0095) comparing IL-2Rα^−/−^ mice to IL-2Rα^+/−^ mice (Figure 1C and D).

### Depletion of IL-17A but not IFN-γ exacerbates autoimmune biliary disease

**Figure 2:**
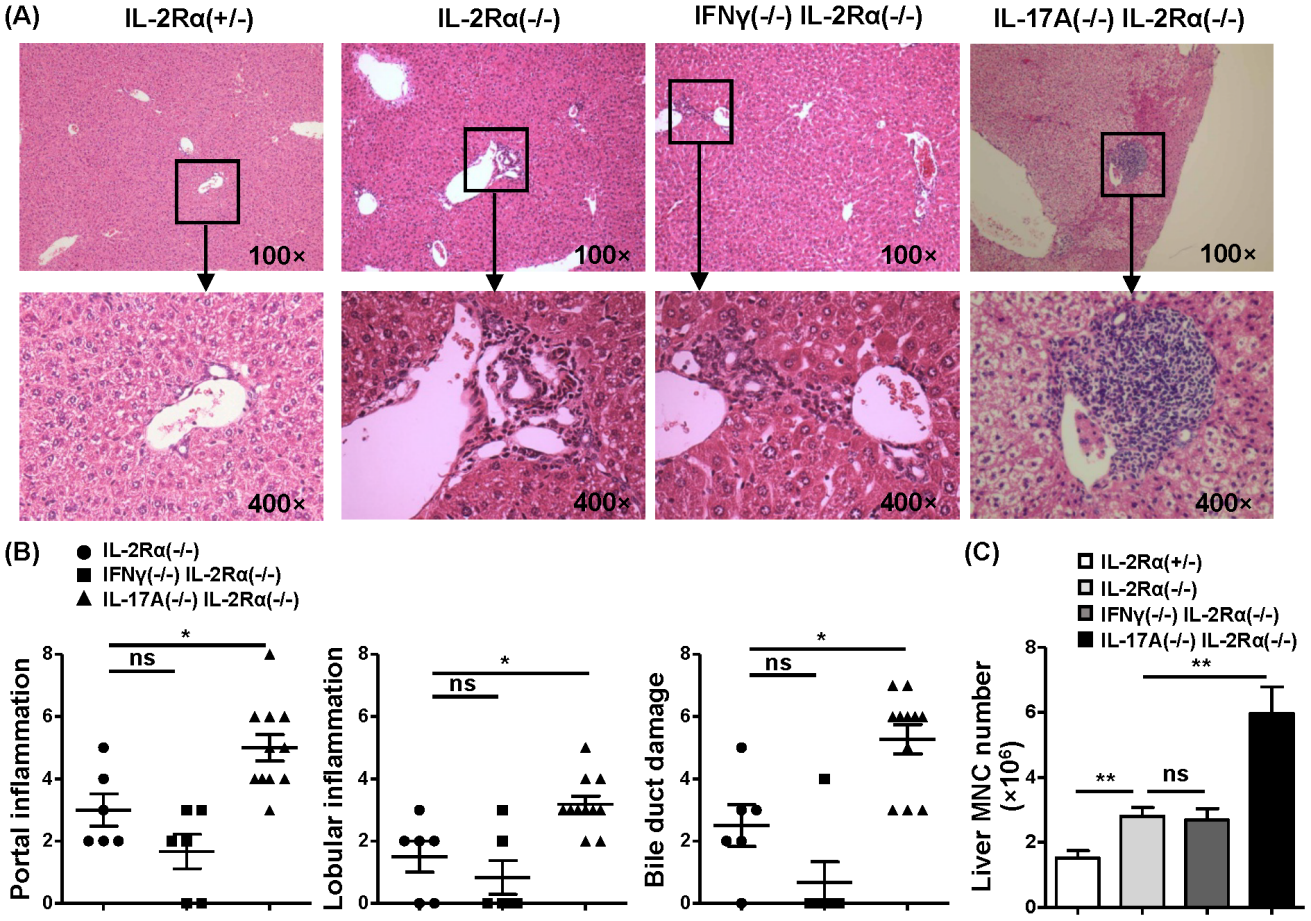
Liver histopathology and mononuclear cell analysis. (A) Representative H&E staining of liver sections. (B) Score of liver portal inflammation, lobular inflammation and bile duct damage of IL-2Rα^−/−^ mice (n = 6), IFN-γ^−/−^IL-2Rα^−/−^ mice (n = 6) and IL-17A^−/−^IL-2Rα^−/−^ mice (n = 11). (C) Total number of mononuclear cells in liver from IL-2Rα^+/−^ (n = 8), IL-2Rα^−/−^ (n = 19), IFN-γ^−/−^IL-2Rα^−/−^ (n = 7), IL-17A^−/−^IL-2Rα^−/−^ mice (n = 17). * P<0.05, ** P<0.01.

To address the roles of IFN-γ and IL-17A on the development of autoimmune biliary disease, we backcrossed IFN-γ knockout mice and IL-17A knockout mice onto IL-2Rα^−/−^ mice respectively to generate IFN-γ^−/−^IL-2Rα^−/−^ mice and IL-17A^−/−^IL-2Rα^−/−^ mice. Histological examination of the liver tissue sections from 12-16-week-old mice demonstrated that IL-17A^−/−^IL-2Rα^−/−^ mice had more lymphocytes infiltrating around portal tracts compared to IL-2Rα^−/−^ mice (*P*<0.05), and more severe lobular inflammation (*P*<0.05). IL-17A^−/−^IL-2Rα^−/−^ mice also had more severe bile duct damage compared to IL-2Rα^−/−^ mice (*P*<0.05). In contrast, there was no significant difference between IFN-γ^−/−^IL-2Rα^−/−^ mice and IL-2Rα^−/−^ mice (Figure 2A, B). We analyzed the total mononuclear cells in liver. Consistent with the liver histopathology, there were more liver infiltrating cells in IL-17A^−/−^IL-2Rα^−/−^ mice compared to IL-2Rα^−/−^ mice (*P* = 0.0058), but no significant difference between IFN-γ^−/−^IL-2Rα^−/−^ mice and IL-2Rα^−/−^ mice (*P* = 0.6720) (Figure 2C). All results suggested that deficiency of IL-17A exacerbated the course of autoimmune cholangitis in IL-2Rα^−/−^ mice.

### CD8+ T cells significantly increased in IL-17A^−/−^IL-2Rα^−/−^ mice

**Figure 3:**
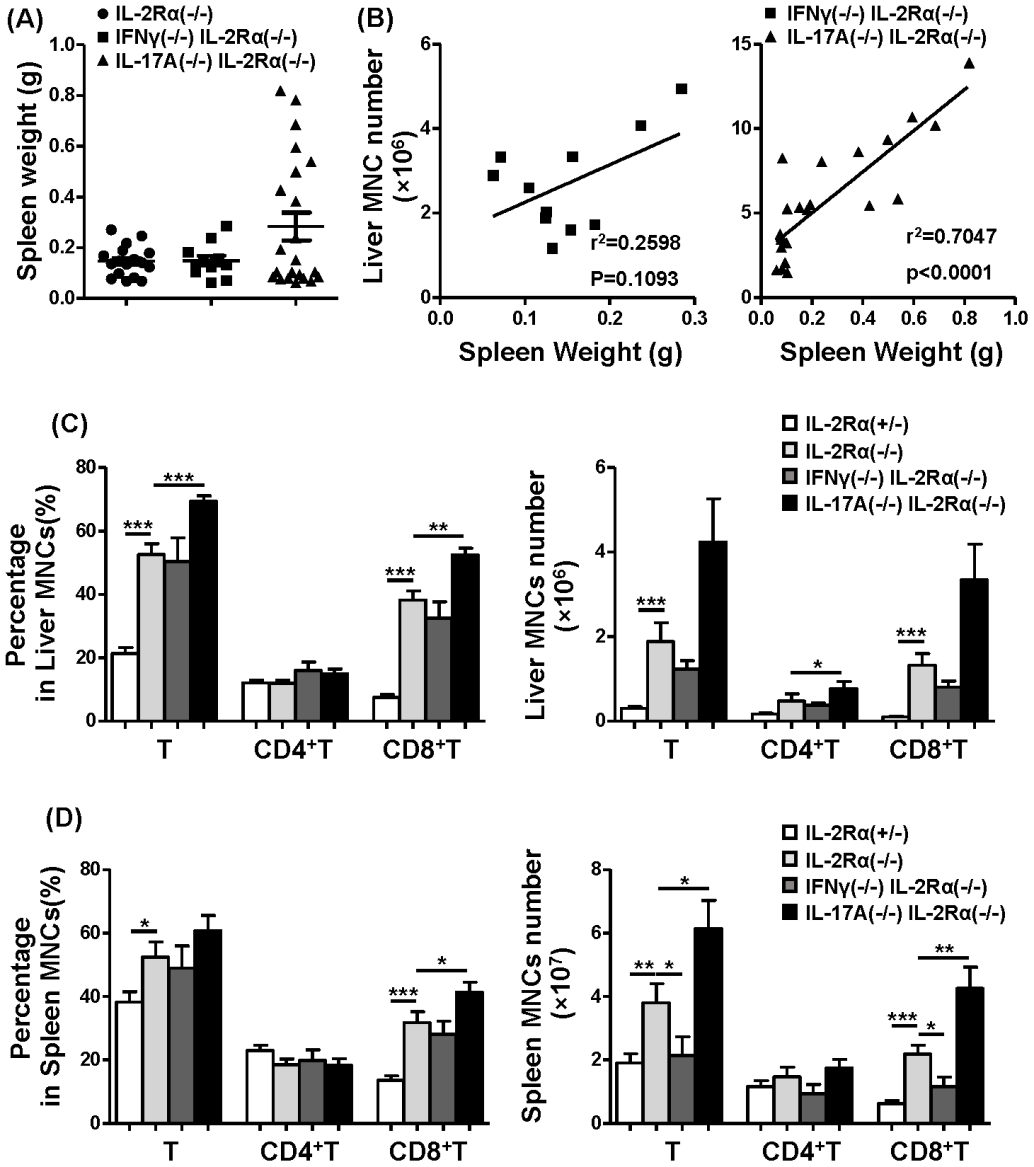
Relationship between splenic weight and liver inflammation. (A) Spleen weight of IL-2Rα^−/−^ (n = 17), IFN-γ^−/−^IL-2Rα^−/−^ (n = 11) and IL-17A^−/−^IL-2Rα^−/−^ mice (n = 22). (B) Correlation between spleen weight and liver MNC in IL-17A^−/−^IL-2Rα^−/−^ and IFN-γ^−/−^IL-2Rα^−/−^ mice. Percentage and absolute number of total T cells (CD3+NK1.1-), CD4+T cells and CD8+T cells from liver (C) and spleen (D) of IL-2Rα^−/−^ (n = 10), IFN-γ^−/−^IL-2Rα^−/−^ (n = 9) and IL-17A^−/−^IL-2Rα^−/−^ mice (n = 10). * P<0.05, ** P<0.01, *** P<0.001.

It has been reported that IL-2Ra^−/−^ mice developed massive enlargement of lymph nodes and spleen [3]. Herein we noted splenomegaly; spleen weight was greater in IL-17A^−/−^IL-2Rα^−/−^ mice. There was also a greater frequency of splenomegaly in IL-17A^−/−^IL-2Rα^−/−^ mice (8/22) compared to IL-2Rα^−/−^ mice (0/17) and IFN-γ^−/−^IL-2Rα^−/−^ mice (0/11) (Figure 3A). The spleen weight of IL-17A^−/−^IL-2Rα^−/−^ mice positively correlated with liver mononuclear cells (Figure 3B). The percentage of total T cells in liver was increased in IL-17A^−/−^IL-2Rα^−/−^ compared to IL-2Rα^−/−^ mice (*P* = 0.0005), due to the remarkable expansion of CD8+T cells (*P* = 0.0011). There were no differences between IFN-γ^−/−^IL-2Rα^−/−^ and IL-2Rα^−/−^ mice. The absolute number of total hepatic T cells (*P* = 0.0524) and CD8+T cells (*P* = 0.0630) trended toward an increase in total hepatic CD4+T cells (*P* = 0.0375) in IL-17A^−/−^IL-2Rα^−/−^ mice compared to IL-2Rα^−/−^ mice, but there were no differences noted in IFN-γ^−/−^IL-2Rα^−/−^ or IL-2Rα^−/−^ mice (Figure 3C). The frequency of splenic CD8+T cells was also significantly increased in IL-17A^−/−^IL-2Rα^−/−^ mice (*P* = 0.0355). The absolute number of splenic CD8+T cell was also increased in IL-17A^−/−^IL-2Rα^−/−^ mice compared with IL-2Rα^−/−^ mice (*P* = 0.0039) (Figure 3D).

### Depletion of IL-17A, but not IFN-γ, ameliorated colitis, and T cells in mesenteric lymph nodes were decreased in IL-17A^−/−^IL-2Rα^−/−^ mice

**Figure 4:**
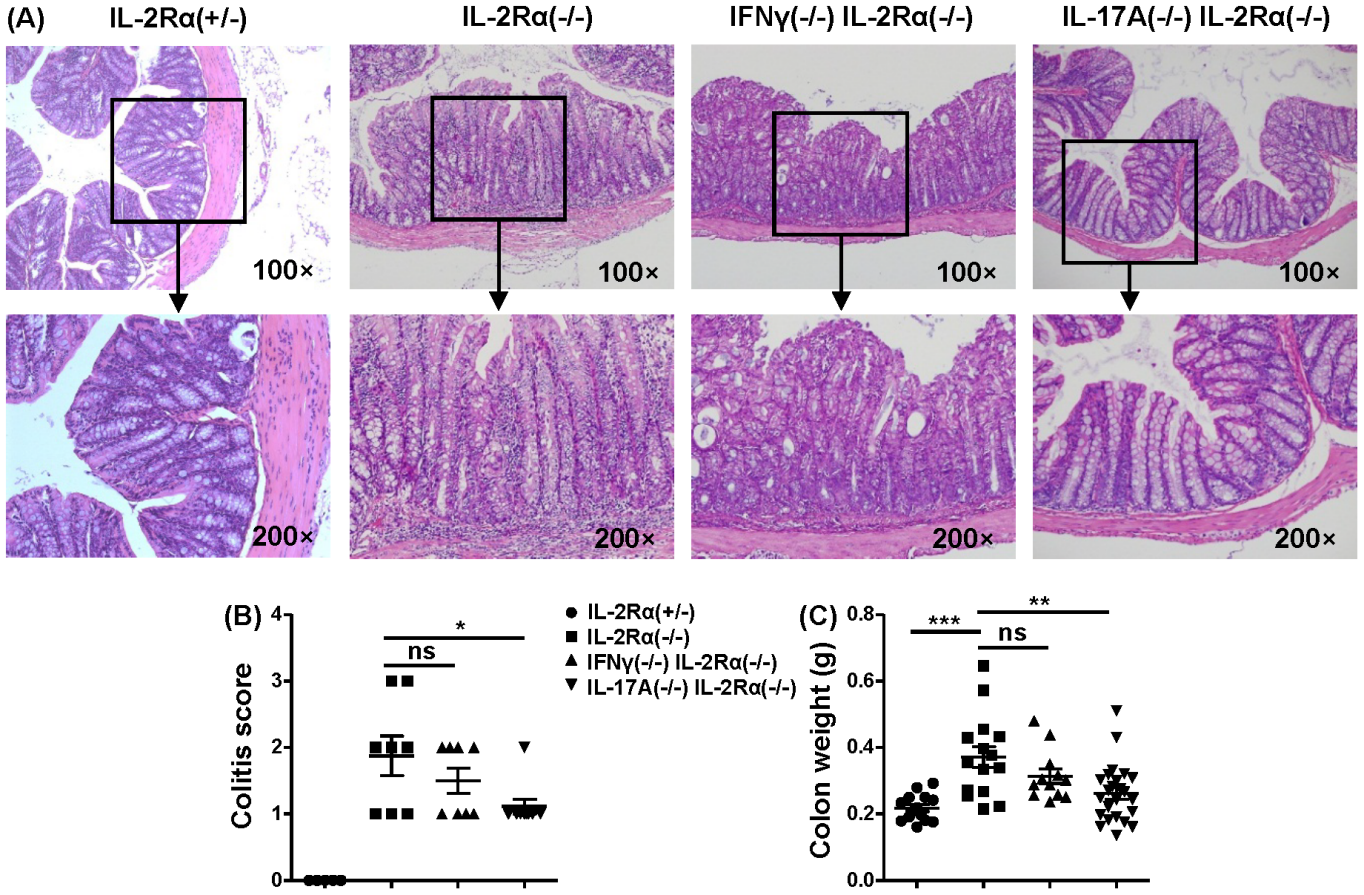
Colitis severity. (A) Representative H&E staining of colon sections from IL-2Rα^+/−^, IL-2Rα^−/−^, IFN-γ^−/−^IL-2Rα^−/−^ and IL-17A^−/−^IL-2Rα^−/−^ mice. (B) Colitis score of IL-2Rα^+/−^ (n = 5), IL-2Rα^−/−^ (n = 8), IFN-γ^−/−^IL-2Rα^−/−^ (n = 8) and IL-17A^−/−^IL-2Rα^−/−^ (n = 9) mice.(C) Colon weight of IL-2Rα^+/−^ (n = 15), IL-2Rα^−/−^ (n = 15), IFN-γ^−/−^IL-2Rα^−/−^ (n = 12) and IL-17A^−/−^IL-2Rα^−/−^ (n = 24) mice. * P<0.05, ** P<0.01, *** P<0.001.

IFN-γ^−/−^IL-2Rα^−/−^ and IL-2Rα^−/−^, but not IL-17A^−/−^IL-2Rα^−/−^ mice frequently suffered from rectal prolapse and diarrhea. Therefore we examined these strains for colitis. There was no significant difference between IFN-γ^−/−^IL-2Rα^−/−^ mice and IL-2Rα^−/−^ mice. Colitis, however, was improved in IL-17A^−/−^IL-2Rα^−/−^ mice compared to IL-2Rα^−/−^ mice (P<0.05), although minimal colitis still occurred in IL-17A^−/−^IL-2Rα^−/−^ mice (Figure 4A and B). Colon weight (which correlates with severity of colitis), was also significantly decreased in IL-17A^−/−^IL-2Rα^−/−^ mice compared to IL-2Rα^−/−^ mice (*P* = 0.0034), but no significant difference was found in IFN-γ^−/−^IL-2Rα^−/−^ mice (*P* = 0.2723) (Figure 4C).

**Figure 5:**
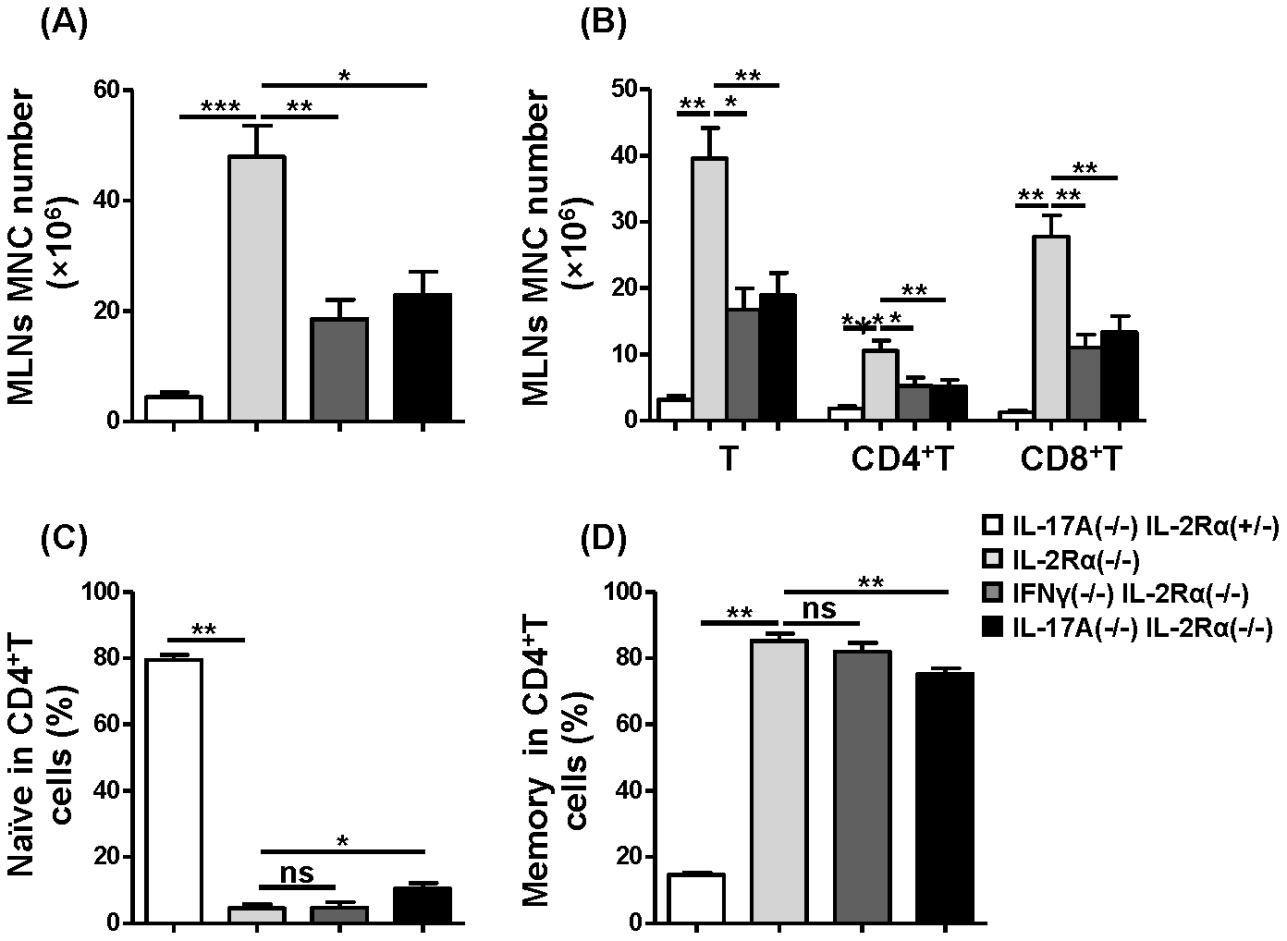
Analysis of IL-2Rα^−/−^ versus IL-17A^−/−^IL-2Rα^−/−^ mesenteric lymph node T cells, CD4+T cells, and CD8+T cells. (A) Total number of mononuclear cells in mesenteric lymph nodes from IL-17A^−/−^IL-2Rα^+/−^ (n = 6), IL-2Rα^−/−^ (n = 9) and IL-17A^−/−^IL-2Rα^−/−^ mice (n = 11).(B) Absolute number of total T cells (CD3+NK1.1-), CD4+T cells and CD8+T cells in mesenteric lymph nodes from IL-17A^−/−^IL-2Rα^+/−^ mice (n = 6), IL-2Rα^−/−^ (n = 9) and IL-17A^−/−^IL-2Rα^−/−^ mice (n = 11). (C, D) Percentage of naïve (C) and memory (D) in CD4+T cells from IL-17A^−/−^IL-2Rα^+/−^ (n = 6), IL-2Rα^−/−^ (n = 7) and IL-17A^−/−^IL-2Rα^−/−^ mice (n = 11). * P<0.05, ** P<0.01, *** P<0.001.

Mesenteric lymphocytes were significantly decreased (*P* = 0.0055) (Figure 5A) in IL-17A^−/−^IL-2Rα^−/−^ mice compared to IL-2Rα^−/−^ mice, as well as the total T cells (*P* = 0.0049), CD4+T cells (*P* = 0.0185) and CD8+T cells (*P* = 0.0039) (Figure 5B). The frequency of naïve CD4+T cells was increased (*P* = 0.0463) (Figure 5C) and the frequency of activated/memory CD4+T cells was significantly reduced in IL-17A^−/−^IL-2Rα^−/−^ mice compared to IL-2Rα^−/−^ mice (*P* = 0.0050) (Figure 5D).

### Cytokines profiles in IL-17A^−/−^IL-2Rα^−/−^ mice

**Figure 6:**
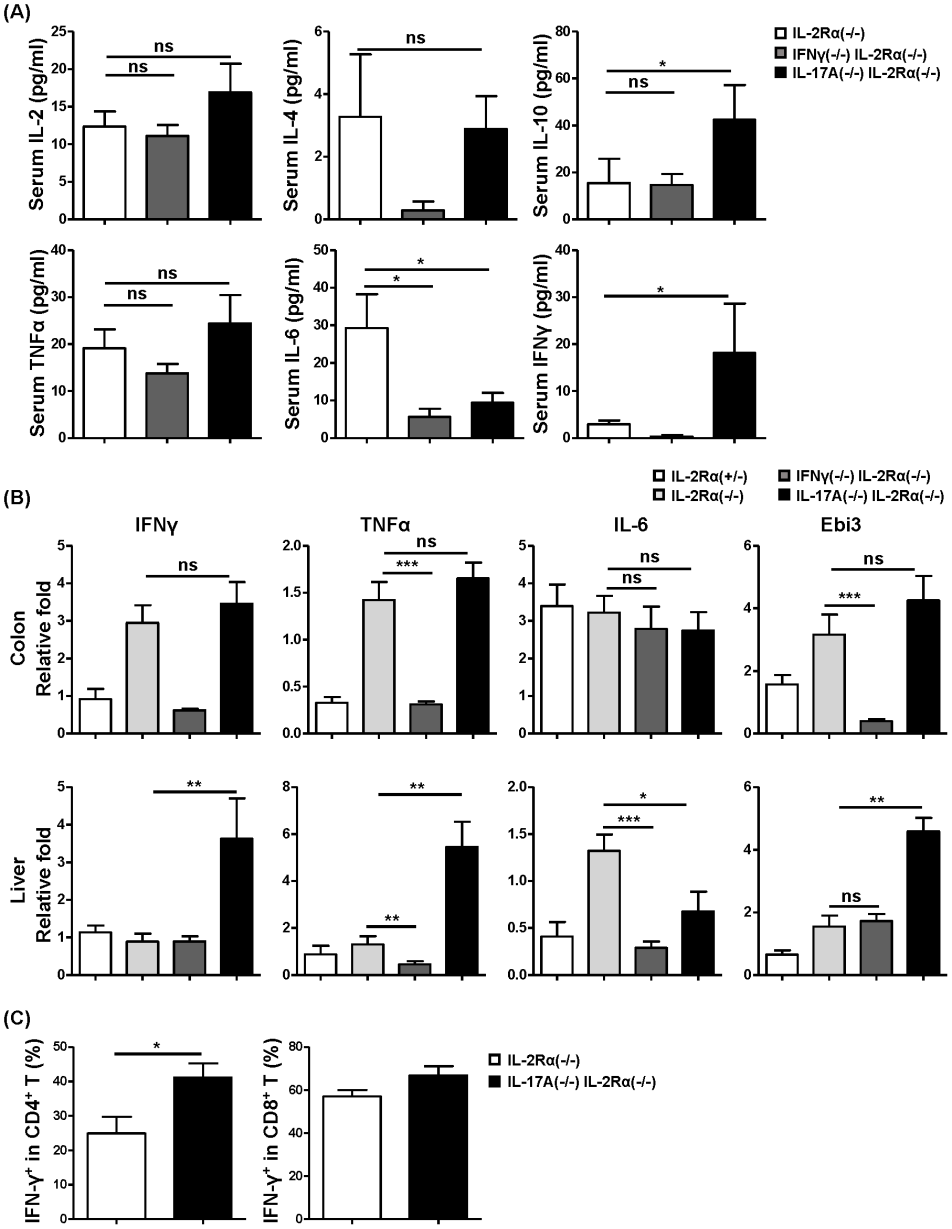
Cytokine analysis. (A) Cytokines in serum samples of IL-2Rα^−/−^ (n = 6) IFN-γ^−/−^IL-2Rα^−/−^ (n = 7) and IL-17A^−/−^IL-2Rα^−/−^ mice (n = 7). (B) Relative levels of cytokine gene mRNA in colon and liver tissues of IL-2Rα^+/−^ (n = 8), IL-2Rα^−/−^ (n = 8), IFN-γ^−/−^IL-2Rα^−/−^ (n = 8) and IL-17A^−/−^IL-2Rα^−/−^ mice (n = 8). (C) Intracellular staining IFN-γ of liver CD4+ T cells and CD8+ T cells from IL-2Rα^−/−^ (n = 7) and IL-17A^−/−^IL-2Rα^−/−^ mice (n = 11). * P<0.05, ** P<0.01, *** P<0.001.

Serum from IL-17A^−/−^IL-2Rα^−/−^ mice compared with IL-2Rα^−/−^ mice contained significantly higher levels of IFN-γ (*P* = 0.0168) and IL-10 (*P* = 0.0444), but lower levels of IL-6 (*P* = 0.0221), and no changes in IL-2 (*P* = 0.3969) or TNF-α (*P* = 0.6019). Meanwhile serum from IFN-γ^−/−^IL-2Rα^−/−^ mice compared with IL-2Rα^−/−^ mice contained lower levels of IL-6 (*P* = 0.0111), and no changes in IL-2 (*P* = 0.5350), IL-10 (*P* = 0.4295) and TNF-α (*P* = 0.2769) (Figure 6A). Further we measured a panel of cytokine mRNA levels in the colon and liver tissue from IFN-γ^−/−^IL-2Rα^−/−^ mice, IL-17A^−/−^IL-2Rα^−/−^ mice and IL-2Rα^−/−^ mice, including IL-7, IL-10, IL-15, IL-18, IL-21, IL-22, IL-17a (data not shown), IL-27 (IL-27p28 and Ebi3), IL-6, TNFα and IFN-γ. As shown in Fig. 6B, deletion of IL-17A resulted in a significantly increased levels of IFN-γ (*P* = 0.0012), TNF-α (*P* = 0.0023) and Ebi3 (*P* = 0.0012), but decreased the level of IL-6 (*P* = 0.0379) in the liver. There were no detectable changes of these cytokines in the colon. And after deletion of IFN-γ in IL-2Rα^−/−^ mice there were lower levels of TNF-α (*P* = 0.0111) and IL-6 (*P* = 0.0006), but no change in Ebi3 (*P* = 0.8048) in the liver. In the colon tissue of IL-2Rα^−/−^ mice, the mRNA levels of TNF-α (*P* = 0.0003) and Ebi3 (*P* = 0.0002) were decreased, whereas that of IL-6 (*P* = 0.2345) reflected no change. There was no change of mRNA levels of IL-17 expression in colon and liver after deletion of IFN-γ in IL-2Rα^−/−^ mice (data not shown). We stimulated hepatic mononuclear cells and stained the cells for intracellular cytokines IFN-γ, IL-4 and IL-17F. The frequency of IFN-γ+ cells was significantly increased both in CD4+T (*P* = 0.0186) cells and CD8+T (*P* = 0.0759) cells from IL-17A^−/−^IL-2Rα^−/−^ mice compared to IL-2Rα^−/−^ mice, although there is only a trend toward significance in CD8+T cells (Figure 6C). Little IL-4 and IL-17F were detected (data not shown).

## Discussion

IL-17A is mainly produced by Th17 cells, also by γδT cells, CD8+T cells, natural killer (NK) cells and NKT cells. The functions of IL-17 are mediated via binding to IL-17RA and IL-17RC, two isoforms of the IL-17 receptor [18]. Th17 cells play an important role in clearing pathogens during host defense reactions and inducing tissue inflammation in some autoimmune diseases while exerting protective effects in other inflammatory conditions. For example, IL-17 plays a proinflammatory effect on the pathogenesis of rheumatoid arthritis [19], [20] and experimental autoimmune encephalomyelitis (EAE) [21]. In studies of graft-versus-host disease (GVHD), IL-17A acts in a protective fashion [22] and may play a role in the pathogenesis of liver pathology. For example, IL-17R is expressed on many types of liver cells including hepatocytes, Kupffer cells, hepatic stellate cells, biliary epithelial cells and sinusoidal endothelial cells [23]–[25]. It has been reported that IL-17 is not involved in the pathogenesis of Con A-induced T-cell hepatitis. In alcoholic liver disease, the number of IL-17+ cells correlates with the liver fibrosis score and lobular inflammation [24]. Moreover, IL-17 also plays an important role in stimulating liver inflammation during HCV and HBV infection [26]–[28]. In IL-2Rα^−/−^ mice, IL-17A levels appear to peak at 8 weeks to 13 weeks of age and then steadily decline, indicating that IL-17 may be involved in earlier disease development [28]. Herein we show that when IL-17A is knocked out in IL-2Rα^−/−^ mice, autoimmune cholangitis is more severe. IL-2Rα^−/−^ mice spontaneously develop clinically apparent autoimmune cholangitis at 8 weeks; the appearance of increased IL-17A, at that time, may serve an anti-inflammatory role.

The role of IL-17 in IBD is controversial. IL-17A plays a proinflammatory role in acute TNBS (Trinitro-Benzene-Sulfonic Acid)-induced colitis [29]. However, in DSS (Dextran Sodium Sulphate)-induced colitis, it acts in a protective fashion [30]. Meanwhile, Rag1^−/−^ hosts transferred with IL-17A^−/−^ CD4+ CD45RB^high^ T cells show more severe colitis compared to Rag1^−/−^ mice received corresponding wild type CD4+ T cells, accompanying by increased IFN-γ level in colon [31]. Thus, IL-17A may modulate Th1 polarization. Transfer of T_reg_ cell–depleted CD4+ CD45RB^low^ cells into Rag^−/−^ mice could also induce colitis, but with a slower onset than with CD4+ CD45RB^high^ T cells. Colitis mediated by CD4+ CD45RB^high^ T cells is characterized by increased Th1 cytokines and T_reg_ cell–depleted CD4+ CD45RB^low^ cells are characterized by an increase in Th17 cytokines [32]. During the course of spontaneously colitis in IL-2Rα^−/−^ mice, the ratio of effector/memory CD4+ T cells significantly increased. Hence, the colitis in IL-2Rα^−/−^ mice may be mediated by CD4+ CD45RB^low^ T cells. The percentage of memory CD4+T cell in mesenteric lymph nodes decreased in IL-17A^−/−^IL-2Rα^−/−^ mice compared to IL-2Rα^−/−^ mice, while there was no change in IFN-γ^−/−^IL-2Rα^−/−^ mice. Previous studies have identified that CD8+T cells mediate intrahepatic damage in IL-2Rα^−/−^ mice. Herein we demonstrate that deletion of IL-17A in IL-2Rα^−/−^ mice is associated with significantly increased intrahepatic CD8+T cells and increased intracellular expression of IFN-γ by CD8+T cells. We examined the IL-7 and IL-15 expression in liver (data not shown), which could promote CD8+T proliferation, but there were no significant differences in mRNA levels for these genes between IL-17A^−/−^IL-2Rα^−/−^ and IL-2Rα^−/−^ mice. These data take on particular significance in the possible use of cytokine blocking reagents to treat either autoimmune cholangitis or colitis. Our data suggest that IL-17A plays a different role in autoimmune cholangitis and colitis and raises concerns and caution regarding the therapeutic use of anti-IL-17A.

IL-17 has pleiotropic effects and the data herein reflect the immunopathology in our unique model. Clearly, further work can potentially explore these relationships between IL-17A and IFN-γ. We emphasize that IL-2Rα^−/−^ mice develop simultaneous cholangitis and colitis. We also suggest that future work explore treatment of mice with established disease using antibodies against IL-17. Our data would further imply the potential of modulating IL-17A in patients with PBC, but also caution against such usage in inflammatory bowel disease. It is likely that the immunopathology in each of these human diseases will be different during different stages of immunopathology and it seems important that any clinical trials include a rigorous analysis of the patient's immune response, including monitoring cytokines. Finally, we should emphasize that this is the first report of the use of this combination of double knockouts as a model to explore autoimmune cholangitis and inflammatory bowel disease. The data, as with any animal model, should be interpreted with caution before any direct extrapolation to human therapy.
